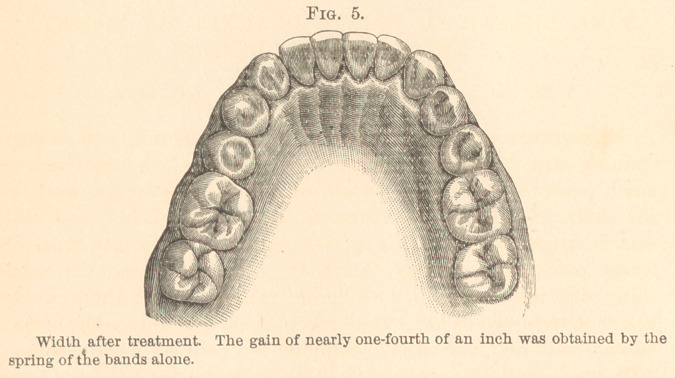# Correcting Irregularities by the Spring of Gold Bands

**Published:** 1890-05

**Authors:** B. S. Byrnes

**Affiliations:** Memphis, Tenn.


### CORRECTING IRREGULARITIES BY THE SPRING OF GOLD BANDS.[1]

BY B. S. BYRNES, D.D.S., MEMPHIS, TENN.

I REGRET to say this mode is yet comparatively new in the profession, although demonstrated thoroughly at the Southern Dental Association, held in New Orleans in the spring of 1885, and published in the *Dental Cosmos* for May, 1886. To-day I know of but few dentists who fully appreciate the merits of the principle.

Dr. Kingsley, in answer to the query as to what kind of fixture he used for regulating teeth, says, "Some variation of an old appliance must be invented for almost every new case." I will go further, for in all of my complicated cases I find that I use one, two, three, and sometimes four variations from my first invention for a certain case, and after passing certain stages will go back to the original appliance; not that the others were failures, for they did their duty as far as they could, but the original would do more, taken from that point, than its substitutes would do if retained; the substitutes did better to pass the work over certain stages than the original could do. To quote Dr. Clark on failures, I will say that I have been unable to obtain the retainer by demanding half of the fee in advance; but will say this much in favor of the principle which I advocate: that all of my patients claim that after wearing the fixture the first day they find their teeth are much more comfortable while the fixtures are on than when they are taken off; for I too "have had them to bring fixtures to me in their pockets, having taken them off the night before to attend a ball."

---

[1] Read at the Union meeting held at Springfield, Mass., October 24, 1889.

In correcting irregularities there are three important points which I keep constantly in mind and labor for faithfully, to accomplish the objects I have in view. First, the more even arrangement of the teeth in the arch; second, the physiognomy, so as to have the features harmonize well; third, and last though not least, the occlusion of the teeth, for thereon depends the success of the case. In following this last point we are simply aiding nature to do a work which she was too feeble to perform when called upon. These three points are likened unto the Holy Trinity, for they are, properly speaking, three in one.

I practise no part of my profession by a fixed rule. I condemn none of the materials used by dentists; neither do I say I never extract a tooth. I study well the nature of each individual case as it presents itself, then follow the dictates of my judgment towards correcting its abnormity.

For illustration, I will give my reasons for treating two cases so entirely different that presented similar appearances of irregularities. The first case, published in the *Cosmos* for May, 1886 (see Fig. 1), shows a prominence of the inferior cuspidata, patient

Fig. 1.

twenty-seven years of age, with wisdom teeth well erupted and crowding all the teeth forward, making the inferior maxillary square and angular at the chin. I at once without hesitancy extracted the first bicuspids, which were perfectly sound, and with simple gold band, on either side embracing first molar, bicuspid, and cuspid, drew the cuspidata back into place until they occluded on the distal surfaces of the superior cuspidata; having reached this stage I had my patient discard the bands. I then propped the teeth apart until the occluding cusps passed each other. (See Fig. 2).

Nature came in and did her work, reducing the angular, protruding chin, and the features are now in perfect harmony; besides

Fig. 2.

overcoming the threatened trismus, the crowded arch has entirely disappeared.

Having treated the first case, I will now introduce the parallel case, which received entirely different treatment. I would not have dreamed of extracting teeth in this case, and why? Because broad, flat features, depressed lips, and a retreating chin presented themselves for treatment as well as irregular teeth. Patient fourteen years of age. I first used a fixture embracing the two

Fig. 3.

Natural width of arch.

bicuspids on either side with a continuous band around the front of the cuspidata and behind the incisors. (See Fig. 3.) The object was to crowd the incisors more together and force them forward. Work progressed well to a certain stage, when my patient complained of the soreness being altogether in the bicuspids. This necessitated another invention.

I at once substituted for the first fixture the following one, which is a simple long strip of gold with the two ends soldered

FIG. 4.

together and woven around the teeth. (See Fig. 4, A.) It was around the first bicuspids on either side, which doubled the strips in a parallel line on the anterior surface of the cuspids; the inner strip was passed behind the incisors and the outer strip was continued on their anterior surface. This simple fixture had a threefold object,—to force the bicuspid outward, to force the incisors forward, and to hold them in line at the same time.

Fixture No. 2 was discarded for two somewhat similar ones. (See Fig. 4, B and C.) The left central being very prominent, I concluded to leave it free; so I placed a small loop around the left lateral, doubling over the cuspid and embracing the first bicuspid on the right side, enclosing lateral and central in the loop. While these two

FIG. 5.

Width after treatment. The gain of nearly one-fourth of an inch was obtained by the spring of the bands alone.

bands were on I found it necessary to weave a band in like manner over the left central, looped from left lateral to right central, which was to bring the incisors in line. (See Fig. 4, D.) Having brought the incisors in line and spread the superior arch by means of occlusion, inasmuch as the bite was very close, I now go back to first principles and make a retaining fixture, which is formed of a simple band embracing the first bicuspid on either side instead of both bicuspids, with a continuous strip passing in front of cuspids and behind incisors. I am only able to pronounce this case a success from the simplicity, ease, and comfort with which the fixtures were worn, as sheer indifference caused my patient to absent herself on one or two occasions for as long a time as two weeks during the process of straightening her teeth.

By way of summary, I will add that the most important point in this case is the improvement of the features, and because it proves a contradiction of my own statement made in New Orleans. When asked what I would do to gain space to bring the teeth into line, I said in such a case I would have to use the jack-screw or coffin-plate, but here I have spread both arches without the screw or plate, and confined my fixtures to the lower teeth exclusively. The patient would not tolerate the rubbers between her teeth that I put in for assistance, knowing they would give less resistance in occluding if I could gain some by that means, yet she wore the bands without complaining.

## Figures and Tables

**Fig. 1. f1:**
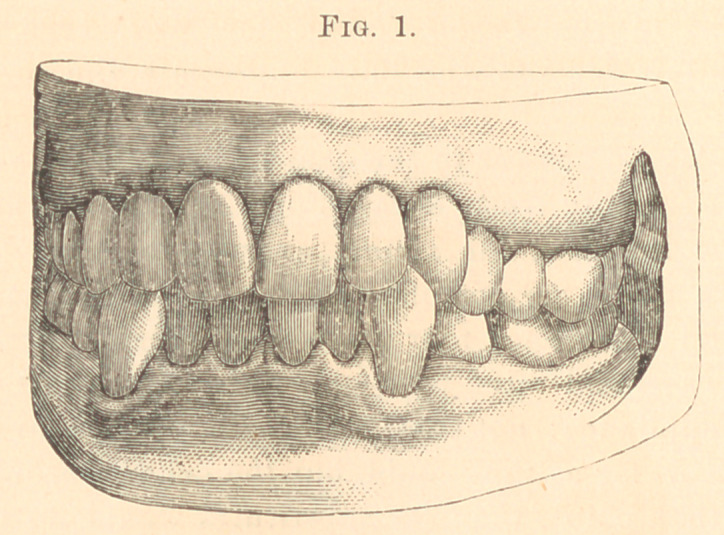


**Fig. 2. f2:**
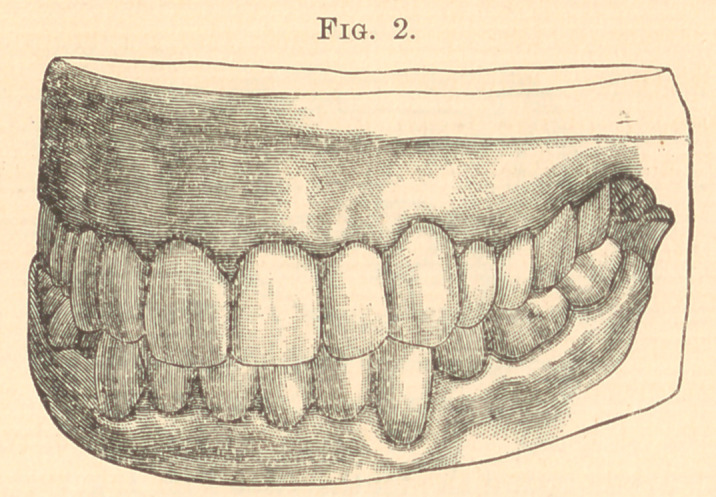


**Fig. 3. f3:**
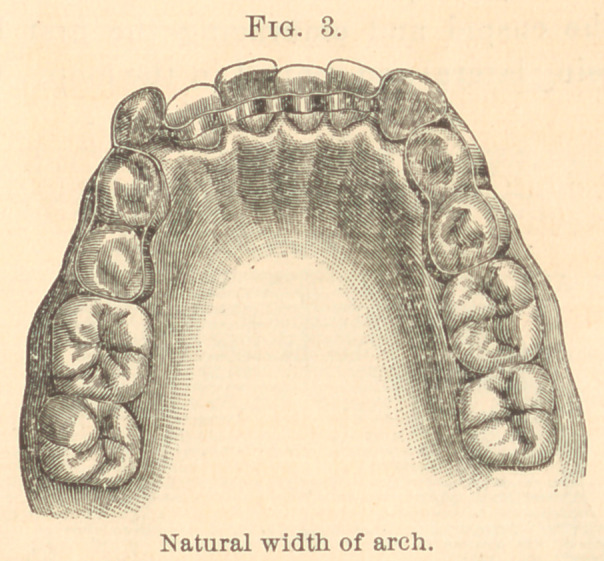


**Fig. 4. f4:**
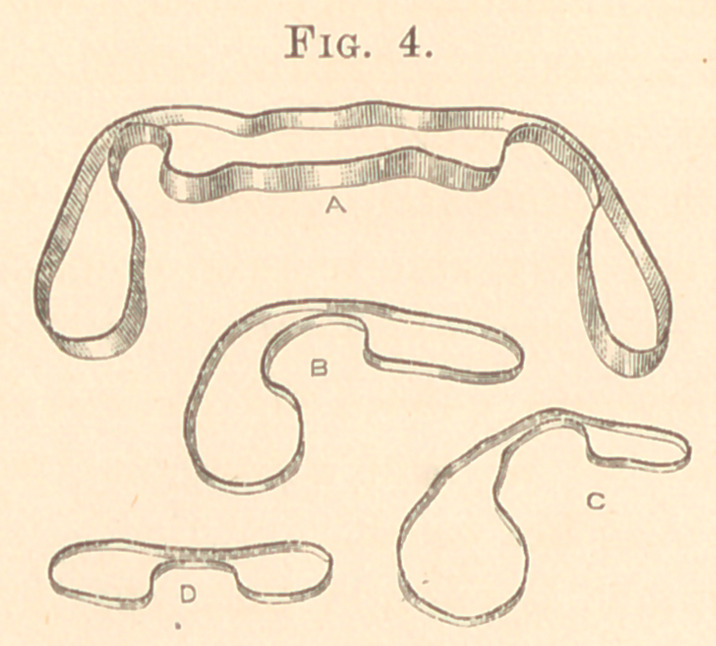


**Fig. 5. f5:**